# A global perspective on bacterial diversity in the terrestrial deep subsurface

**DOI:** 10.1099/mic.0.001172

**Published:** 2023-01-17

**Authors:** A. Soares, A. Edwards, D. An, A. Bagnoud, J. Bradley, E. Barnhart, M. Bomberg, K. Budwill, S. M. Caffrey, M. Fields, J. Gralnick, V. Kadnikov, L. Momper, M. Osburn, A. Mu, J. W. Moreau, D. Moser, L. Purkamo, S. M. Rassner, C. S. Sheik, B. Sherwood Lollar, B. M. Toner, G. Voordouw, K. Wouters, A. C. Mitchell

**Affiliations:** ^1^​ Department of Geography and Earth Sciences (DGES), Aberystwyth University (AU), Aberystwyth, UK; ^2^​ Institute of Biology, Environmental and Rural Sciences (IBERS), AU, Aberystwyth, UK; ^3^​ Department of Plant and Microbial Biology, University of Minnesota, Minneapolis, MN, USA; ^4^​ Interdisciplinary Centre for Environmental Microbiology (iCEM), AU, Aberystwyth, UK; ^5^​ Department of Biological Sciences, University of Calgary, Calgary, Canada; ^6^​ Institut de Génie Thermique (IGT), Haute École d'Ingénierie et de Gestion du Canton de Vaud (HEIG-VD), Yverdon-les-Bains, Switzerland; ^7^​ School of Geography, Queen Mary University of London, London, UK; ^8^​ U.S. Geological Survey (USGS), USA, Reston, VA, USA; ^9^​ Center for Biofilm Engineering (CBE), Montana State University, Bozeman, MT, USA; ^10^​ VTT Technical Research Centre of Finland, Finland; ^11^​ Alberta Innovates, Canada; ^12^​ University of Toronto, Toronto, Canada; ^13^​ Department of Microbiology & Immunology, MSU, Bozeman, MT, USA; ^14^​ Institute of Bioengineering, Research Center of Biotechnology, Russian Academy of Sciences, Russia; ^15^​ Department of Earth, Atmospheric and Planetary Sciences (DEAPS), The Massachusetts Institute of Technology (MIT), Cambridge, MA, USA; ^16^​ Department of Earth and Planetary Sciences, Northwestern University, Evanston, IL, USA; ^17^​ Department of Microbiology and Immunology at the Peter Doherty Institute for Infection and Immunity, University of Melbourne, Melbourne, Australia; ^18^​ Doherty Applied Microbial Genomics, Department of Microbiology and Immunology at the Peter Doherty Institute for Infection and Immunity, University of Melbourne, Melbourne, Australia; ^19^​ Microbiological Diagnostic Unit Public Health Laboratory, Department of Microbiology and Immunology, University of Melbourne, Melbourne, Australia; ^20^​ School of Earth Sciences, The University of Melbourne, Parkville, Australia; ^21^​ Division of Hydrologic Sciences, Desert Research Institute (DRI), Las Vegas, NV, USA; ^22^​ School of Earth and Environmental Sciences (SEES), University of St. Andrews, St. Andrews, UK; ^23^​ Geological Survey of Finland (GTK), Finland; ^24^​ Large Lakes Observatory, University of Minnesota, Duluth, MN, USA; ^25^​ Department of Earth Sciences, University of Toronto, Toronto, Canada; ^26^​ Department of Soil, Water & Climate, University of Minnesota, Minneapolis/Saint Paul, MN, USA; ^27^​ Institute for Environment, Health and Safety (EHS), Belgian Nuclear Research Centre SCK•CEN, Mol, Belgium; ^†^​ Present address: Group for Aquatic Microbial Ecology (GAME), University of Duisburg-Essen, Campus Essen - Environmental Microbiology and Biotechnology, Universitätsstr. 5, 45141 Essen, Germany

**Keywords:** deep subsurface, 16S rRNA gene, meta-analysis, bacterial

## Abstract

While recent efforts to catalogue Earth’s microbial diversity have focused upon surface and marine habitats, 12–20 % of Earth’s biomass is suggested to exist in the terrestrial deep subsurface, compared to ~1.8 % in the deep subseafloor. Metagenomic studies of the terrestrial deep subsurface have yielded a trove of divergent and functionally important microbiomes from a range of localities. However, a wider perspective of microbial diversity and its relationship to environmental conditions within the terrestrial deep subsurface is still required. Our meta-analysis reveals that terrestrial deep subsurface microbiota are dominated by *Betaproteobacteria, Gammaproteobacteria* and *Firmicutes *, probably as a function of the diverse metabolic strategies of these taxa. Evidence was also found for a common small consortium of prevalent *Betaproteobacteria* and *Gammaproteobacteria* operational taxonomic units across the localities. This implies a core terrestrial deep subsurface community, irrespective of aquifer lithology, depth and other variables, that may play an important role in colonizing and sustaining microbial habitats in the deep terrestrial subsurface. An *in silico* contamination-aware approach to analysing this dataset underscores the importance of downstream methods for assuring that robust conclusions can be reached from deep subsurface-derived sequencing data. Understanding the global panorama of microbial diversity and ecological dynamics in the deep terrestrial subsurface provides a first step towards understanding the role of microbes in global subsurface element and nutrient cycling.

## Data Summary

The 16S rRNA gene sequencing data utilized in the present study are available on NCBI under the following project accessions: PRJNA262938, PRJNA268940, PRJNA248749, PRJNA251746, PRJNA375701, PRJEB1468 and PRJEB10822. The code used for the processing and data analysis of the datasets is available at: https://github.com/GeoMicroSoares/mads_scripts.

## Introduction

Understanding the distribution of microbial diversity is pivotal for advancing our knowledge of deep subsurface global biogeochemical cycles [1, 2]. Subsurface biomass is suggested to have exceeded that of the Earth’s surface by an order of magnitude (~45 % of Earth’s total biomass) before land plants evolved, ca. 0.5 billion years ago [3]. Integrative modelling of cell count and quantitative PCR (qPCR) data and geophysical factors indicated in late 2018 that the bacterial and archaeal biomass found in the global deep subsurface may range from 23 to 31 petagrams of carbon (PgC) [4]. These values halved estimates from efforts earlier that year but maintained the notion that the terrestrial deep subsurface holds ca. 5-fold more bacterial and archaeal biomass than the deep marine subsurface [4, 5]. Further, it is expected that 20–80 % of the possible 2–6×10^29^ prokaryotic cells present in the terrestrial subterranean biome exist as biofilms and play crucial roles in global biogeochemical cycles [4, 6, 7].

Cataloguing microbial diversity and functionality in the terrestrial deep subsurface has mostly been achieved by means of marker gene and metagenome sequencing from aquifers associated with coals, sandstones, carbonates and clays, as well as deep igneous and metamorphic rocks [8–19]. Only recently has the first comprehensive database of 16S rRNA gene-based studies targeting terrestrial subsurface environments been compiled [4]. This work focused on updating estimates for bacterial and archaeal biomass, and cell numbers across the terrestrial deep subsurface, but also linked the identified bacterial and archaeal phylum-level compositions to host-rock type, and to 16S rRNA gene region primer targets [4]. While highlighting *Firmicutes* and Proteobacterial dominance in the bacterial component of the terrestrial deep subsurface, no further taxonomic insights emerged. Genus-level identification remains an important niche necessary for understanding community composition, inferred metabolism and hence microbial contributions of distinct community members to biogeochemical cycling in the deep subsurface [15, 20–22]. Indeed, such genus-specific traits have been demonstrated to be critical for understanding crucial biological functions in other microbiomes, and genus-specific functions of relevance for deep subsurface biogeochemistry are clear [23–25].

So far, the potential biogeochemical impacts of microbial activity in the deep subsurface have been inferred through shotgun metagenomics, as well as from incubation experiments of primary geological samples amended with molecules or minerals of interest [16, 17, 19, 26–29]. Recent studies of deep terrestrial subsurface microbial communities further suggest that these are metabolically active, often associated with novel uncultured phyla, and potentially directly involved in carbon and sulphur cycling [30–36]. Concomitant advancements in subsurface drilling, molecular methods and computational techniques have aided exploration of the subsurface biosphere, but serious challenges remain, mostly related to deciphering sample contamination by drilling methods, community interactions with reactive casing materials and sample transportation to laboratories for processing [37, 38]. The logistical challenges inherent in accessing and recovering *in situ* samples from hundreds to thousands of metres below the surface complicate our view of terrestrial subsurface microbial ecology [39].

In this study, we capitalize on the increased availability of 16S rRNA gene amplicon data from multiple studies of the terrestrial deep subsurface conducted over the last decade. We apply bespoke bioinformatics scripts to generate insights into the microbial community structure and controls upon bacterial microbiomes of the terrestrial deep subsurface across a large distribution of habitat types on multiple continents. The deep biosphere is as-yet undefined as a biome – elevated temperature, anoxic conditions, varying levels of organic carbon, and measures of isolation from the surface photosphere are some of the criteria used, albeit without a consensus. For this work a more general approach has been taken to define the terrestrial deep subsurface for the purposes of this initial examination as the zone at least 100 m from the surface [7, 40, 41].

## Methods

### Data acquisition

The Sequence Read Archive database of the National Center for Biotechnology Information (SRA-NCBI) was queried for 16S rRNA-based deep subsurface datasets (excluding marine and ice samples, as well as any human-impacted samples); available studies were downloaded using the SRA Run Selector. Studies were selected considering their metadata and information on sequencing platform used – i.e. only samples derived from 454 pyrosequencing and Illumina sequencing were considered. Due to a lack of public availability for Illumina datasets targeting environments of interest, only 454 pyrosequencing datasets were retained. Analysis of related literature resulted in the detection of other deposited studies that previous search efforts in NCBI-SRA failed to detect. Further private contacts allowed access to unpublished data included in this study. The final list of NCBI accession numbers**,** totalling 222 samples, was downloaded using *fastq-dump* from the SRA toolkit (https://hpc.nih.gov/apps/sratoolkit.html)

As seen in Table 1, required metadata included host-rock lithology, general and specific geographical locations, depth of sampling, DNA extraction method, sequenced 16S rRNA gene region and sequencing method. Any samples for which the above-mentioned metadata could not be found were discarded and not considered for downstream analyses.

**Table 1.** Metadata table for the studies utilized in this meta-analysis (see Table S3, Fig. S3 for more details) na, Not available. The dataset unavailable through SRA is available in JGI GOLD Study ID Gs0047444.

SRA accession	DOI	Year(s) of sampling	Final no. of samples	Location	Depth gradient (m)	Host rock	Final no. of sequences	Final no. of OTUs	Reference
PRJNA262938	10.3389/fmicb.2014.00610	2013/2014	6	South Dakota, USA	243.84–1478.28	Sulphide-rich schists	170 364	1367	[50]
PRJNA268940	na	2007–2011	8	California, USA; Ontario, Canada	94–2383	Dolomite, tuff, rhyolite/tuff-breccia	68 071	741	na
PRJNA248749	na	2011	6	Minnesota, USA	730	Haematite iron formation	69 757	511	na
PRJNA251746	na	2009	6	Washington, USA	393–1135	Basalt	30 121	613	na
PRJNA375701	10.1016 /j.coal.2016.05.001	2013	4	Montana, USA	109–114.7	Sub-bituminous coal, shale, sandstone, siltstone	35 926	154	[51]
na	10.1128/AEM.01737–15	2009	27	Alberta, Canada	140–1064.4	Sub-bituminous coal, volatile bituminous coal	100 618	5110	[52]
PRJEB1468	10.1111/1574–6941.12171	na	6	Mol, Belgium	217–232	Kaolinite/illite and smectite	47 123	497	[27]
PRJEB10822	10.5194/bg-13-3091-2016	2009–2011	7	Outokumpu, Finland	180–2300	Mica-schist, biotite-gneiss, chlorite–sericite-schist	12 290	177	[11]

### Pre-processing of 16S rRNA gene datasets

A customized pipeline was created in *bash* language making use of *python* scripts developed for QIIME v1.9.1, to facilitate bioinformatic analyses in this study (see https://github.com/GeoMicroSoares/mads_scripts for scripts) [42]. Briefly, demultiplexed FASTQ files were processed to create an operational taxonomic unit (OTU) table. Quality control steps involved trimming, quality-filtering and chimera checking by means of USEARCH 6.1 [43]. Sequence data that passed quality control were then subjected to closed-reference (CR) OTU-picking on a per-study basis using UCLUST and reverse strand matching against the silva v123 taxonomic references (https://www.arb-silva.de/documentation/release-123/) [43]. CR OTU picking excludes OTUs whose taxonomy has not been found in the 16S rRNA gene database used. Although this limits the recovery of prokaryotic diversity to that recorded in the database, cross-study comparisons of bacterial communities generated by different 16S rRNA gene primers are made possible. This conservative approach classified OTUs in each study individually to the common 16S rRNA gene reference database from the merging of all classification outputs. A single BIOM (Biological Observation Matrix) file was generated using QIIME’s *merge_otu_tables.py* script. The BIOM file was then filtered to exclude samples represented by fewer than two OTUs using *filter_samples_from_otu_table.py,* as well as OTUs represented by one sequence (singleton OTUs) by using *filter_otus_from_otu_table.py*. In an attempt to reduce the impacts of potential contaminant OTUs from the dataset, the post-singleton filtered dataset was further filtered to include only OTUs represented by at least 500 sequences and present in at least 10 samples overall using *filter_otus_from_otu_table.py*.

### Data analysis

All downstream analyses were conducted using the *phyloseq* (https://github.com/joey711/phyloseq) package within R, which allowed for simple handling of metadata and taxonomy and abundance data [44–46]. Merged and filtered BIOM files were imported into R using internal *phyloseq* functions, which allowed further filtering, transformation and plotting of the dataset (see https://github.com/GeoMicroSoares/mads_scripts for scripts). Briefly, following a general assessment of the number of reads across samples and OTUs, *tax_glom* (*phyloseq*) allowed the agglomeration of the OTU table at the phylum level. For the metadata category-directed analyses, the function *merge_samples* (*phyloseq*) created averaged OTU tables, which permitted testing of hypotheses for whether geology or depth had significant impacts on bacterial community structure and composition. Computation of a Jensen–Shannon divergence PCoA (principal coordinate analysis) was achieved with ordinate (*phyloseq*), which makes use of metaMDS (*vegan*) [47, 48]. All figures were plotted via the *ggplot2* R package (https://github.com/tidyverse/ggplot2), except for the UpsetR plot in Fig. S4, which was plotted with the package *UpsetR* (https://github.com/hms-dbmi/UpSetR).

## Results

A total of 233 publicly available subsurface samples targeting multiple 16S rRNA gene hypervariable regions originating in nine countries were originally downloaded from the NCBI SRA database. These accounted for 24 632 035 chimera-checked sequences [11, 27, 49–53], which underwent silva 123-aided CR OTU-picking. The discovery of 46 OTUs classified as Chloroplast (* Cyanobacteria *) and phototrophic members of the phyla *Chloroflexi* and *Chlorobi* as well as orders *Rhodospirillales* and *Chromatiales* (*Alpha*- and *Gammaproteobacteria* classes, respectively) justified the use of additional stricter contamination-aware filtering (see Methodology, Table S1 for differences in numbers of reads between methods).

The final dataset consisted of 70 samples and 2207 OTUs (513 929 sequences). Seventeen aquifers were included that were associated with either sedimentary- or crystalline-host rocks, from depths spanning 94–2300 m below the land surface, targeting mostly groundwater across five countries (Table S2). Nine DNA extraction techniques were used in these studies, ranging from standard and modified kit protocols (e.g. MOBIO PowerSoil) to phenol–chloroform and CTAB/NaCl-based methods [50, 51, 54–57]. Six different primer pair amplified regions of the 16S rRNA gene with 454 pyrosequencing technology were used to generate the datasets (see Fig. S1). Metadata variables that were unavailable for all samples in the dataset were excluded from the statistical analyses. All studies followed aseptic sample handling protocols and included DNA extraction and PCR controls (for further information see Methods sections of the papers enumerated in Table 1) as per recommended guidelines for the subsurface microbiology community [38, 58].

**Fig. 1.**
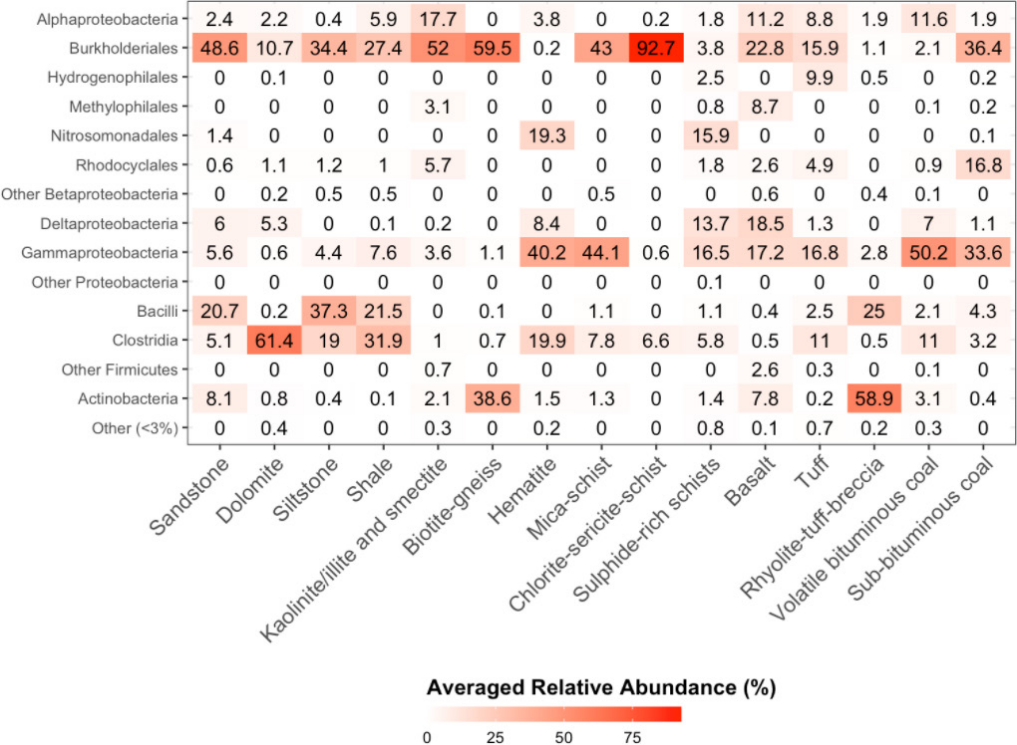
Averaged relative abundances (coloured by increasing percentage abundance) of the most abundant taxonomic groups (*y*-axis) across the dataset across all analysed aquifer lithologies (*x*-axis).

Among a total of 45 detected bacterial phyla, *Proteobacteria* were seen to dominate most deep subsurface community profiles in this dataset (Fig. 1). The most abundant proteobacterial classes (*Alpha*-, *Beta*-, *Delta*-, *Gammaproteobacteria *) represented 57.2 % of the total number of reads. *Betaproteobacteria *, chiefly represented by the order *Burkholderiales *, accounted for 26.1 % of all reads in the dataset. The order *Burkholderiales* was the main component of some host-rocks, accounting for up to 59.5 and 92.7 % of host-rock-level relative abundance profiles for biotite-gneiss and chlorite-sericite-schist (see Fig. S2 for standard deviations of Fig. 1) and co-dominated others. *Gammaproteobacteria* and *Clostridia* (* Firmicutes *) were key components of other profiles. *Clostridia* and other *Firmicutes* accounted for large fractions of sedimentary host-rocks (dolomite, siltstone and shale) and a haematite iron formation. Finally, *Actinobacteria* was the most abundant taxonomic group in rhyolite-tuff-breccia.

**Fig. 2.**
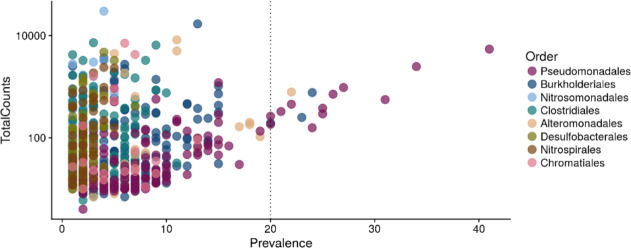
Prevalence (number of samples in which an OTU is present, *x*-axis) of OTUs across the dataset and associated reads (*y*-axis). Colours depict classification of OTUs at the order level. The vertical line is at 20 samples on the *x*-axis to highlight OTUs present in 20 or more samples.

Analysis of prevalence across the dataset revealed that seven OTUs, all affiliated with the genus *Pseudomonas *, were present in more than 25 and up to 41 samples, accounting for 18 149 reads (3.5 % of the total reads, see Fig. 2, Table S3). Other bacterial orders, namely *Burkholderiales *, *Alteromonadales* and *Clostridiales* (* Betaproteobacteria *, *Gammaproteobacteria *, *Clostridia *) were also highly prevalent throughout. Network analysis (Table 2) highlighted a *Pseudomonas* OTU highly connected to other OTUs in the dataset. Furtherore, blast results indicated that recovered sequences for OTUs affiliated with this genus were generally associated with marine and terrestrial soil and sediments (see Fig. S3, Table S4) [59]. Four OTUs affiliated to *Burkholderiales* (* Betaproteobacteria *)*,* the second most prevalent order in the dataset, were also found to be connected to up to 34 other OTUs. The genus *Thauera* (*Betaproteobacteria, Rhodocyclales*), represented by a single OTU, was the second most central to the dataset.

**Table 2.** Top 10 most central OTUs (Proteobacterial classes highlighted) in the Jaccard distances network (as defined by eigenvector centrality scores, or the scored value of the centrality of each connected neighbour of an OTU) and corresponding closeness centrality (scores of shortest paths to and from an OTU to all the remaining OTUs in a network) and degree (number of directly connected edges, or OTUs) values

OTU ID	OTU classification	Centrality	Closeness	Degree
EF554871.1.1486	*Proteobacteria;* ***Gammaproteobacteria*** *; Pseudomonadales; Pseudomonadaceae; Pseudomonas*	1.0000000	2.13e-05	38
HH792638.1.1492	*Proteobacteria;* ***Betaproteobacteria*** *; Rhodocyclales; Rhodocyclaceae; Thauera*	0.9753542	2.13e-05	36
HQ681977.1.1496	*Proteobacteria;* ***Betaproteobacteria*** *; Burkholderiales; Comamonadaceae; Diaphorobacter*	0.9445053	2.13e-05	34
KF465077.1.1336	*Proteobacteria;* ***Betaproteobacteria*** *; Burkholderiales; Comamonadaceae; Acidovorax*	0.8887751	2.13e-05	30
JQ072853.1.1348	*Proteobacteria;* ***Betaproteobacteria*** *; Rhodocyclales; Rhodocyclaceae; Thauera*	0.8808435	2.13e-05	30
KM200734.1.1449	*Proteobacteria;* ***Alphaproteobacteria*** *; Rhizobiales; Rhizobiaceae; Rhizobium*	0.8716886	2.13e-05	31
KC758926.1.1392	*Proteobacteria;* ***Betaproteobacteria*** *; Burkholderiales; Comamonadaceae; Acidovorax*	0.8662805	2.13e-05	29
FJ032194.1.1456	*Proteobacteria;* ***Betaproteobacteria*** *; Burkholderiales; Comamonadaceae; Rhodoferax*	0.8662805	2.13e-05	29
EU771645.1.1366	*Firmicutes; Bacilli; Bacillales; Planococcaceae; Planomicrobium*	0.8476970	2.13e-05	30
JN245782.1.1433	*Proteobacteria;* ***Alphaproteobacteria*** *.; Rhodobacterales; Rhodobacteraceae; Defluviimonas*	0.8356655	2.13e-05	29

While relative abundance patterns across the dataset (Fig. 1) indicate that lithology could influence microbial community composition and structure, sample sizes for each host-rock in the final dataset were insufficient to provide robust statistical support of that hypothesis. Despite this, host-rocks (10 out of 15) presented, on average, more unique OTUs than they shared with other host-rocks (Fig. S4). In particular, in sulphide-rich schists, 73 % of the OTUs were, on average, unique to the host-rock. Sub-bituminous and volatile bituminous coals shared a total of 143 OTUs; this was the strongest interaction between host-rocks in the dataset. No significant correlations were found for the presence of the most abundant clades in the dataset and depth, *Actinobacteria* being the only major taxonomic group to have a positive, albeit weak, correlation with depth (Pearson’s *r*=0.42, *P*<0.01, Fig. S5). Proportions of *Beta*- and *Gammaproteobacteria* generally decreased with depth (Pearson’s *r*=−0.29 and −0.093, respectively), but no other major clades were shown to correlate.

Ordination of the final dataset further suggests 50.6 % of Jensen–Shannon distances were significantly explained by aquifer lithology (ADONIS/PERMANOVA, F-statistic=4.65, *P*<0.001, adjusted Bonferroni correction *P*<0.001). Other environmental features such as absolute depth and medium-scale location (i.e. state, region of the sampling site) explained only 3.08 and 2.78 % of the significant metadata-driven variance in bacterial community structure, respectively (ADONIS/PERMANOVA, F-statistic=3.95, 3.57, *P*<0.001, adjusted Bonferroni correction *P*<0.001). Finally, no evidence was found for DNA extraction or 16S rRNA gene region significantly affecting bacterial community structure in this meta-analysis (ADONIS/PERMANOVA, F-statistic=3.85, 3.23, *P*<0.01, adjusted Bonferroni correction *P*<0.001).

## Discussion

The deep biosphere is an active, diverse biome still largely under-investigated in terms of the Earth’s biogeochemistry [12, 60–62]. In this study, publicly available 16S rRNA gene data revealed a prevalence of *Betaproteobacteria* and *Gammaproteobacteria* in the deep biosphere that may be explained by the diverse metabolic capabilities of taxa within these clades. The families *Gallionellaceae, Pseudomonadaceae*, *Rhodocyclaceae* and *Hydrogeniphillaceae* within *Betaproteobacteria* and *Gammaproteobacteria* are suggested to play critical roles in deep subsurface iron, nitrogen, sulphur and carbon cycling across the world [50, 61, 63]. The relative abundance of the order *Burkholderiales* (* Betaproteobacteria *) in surficial soils has previously been correlated (*R* ^2^=0.92, ANOVA *P*<0.005) with mineral dissolution rates, while the genus *Pseudomonas* (* Gammaproteobacteria *) is widely known to play a key role in hydrocarbon degradation, denitrification and coal solubilization in different locations [64–66]. The dominance of *Betaproteobacteria* and *Gammaproteobacteria* in coals builds on culture-based evidence of widespread degradation of coal-associated complex organic compounds by these classes [67–70].

The metabolic plasticity of the orders *Pseudomonadales* and *Burkholderiales* has been demonstrated and may be a catalyst for their apparent centrality across the terrestrial deep subsurface microbiomes analysed in this study [71–74]. These bacterial orders may represent important keystone taxa in microbial consortia responsible for providing critical substrates to other colonizers in deep subsurface environments [75, 76]. In particular, given the number of highly central *Pseudomonas *-affiliated OTUs and the prevalence of this genus in the dataset, we suggest that this genus may play a central role in establishing conditions for microbial colonization in many terrestrial subsurface environments. The genus *Pseudomonas* and possibly several members of *Burkholderiales* may therefore comprise an important component of the global core terrestrial deep subsurface bacterial community [11]. Geographically comprehensive RNA-based approaches should in the future investigate the potential roles of the genus *Pseudomonas* and order *Burkholderiales* in this biome.

The class *Clostridia* was found to be prevalent across the dataset and to dominate in sedimentary host-rocks (dolomite, siltstone and shale) in this study. This class includes anaerobic hydrogen-driven sulphate reducers also known to sporulate and metabolize a wide range of organic carbon compounds [77]. Previously, members of *Clostridia* have also been identified as dominant components in extremely deep subsurface ecosystems beneath South Africa, Siberia and California (USA) from metabasaltic and metasedimentary lithologies [78]. Adaptation to extreme environments in this class has been associated with diverse metabolic capabilities that include sporulation ability and capacity for CO_2_- or sulphur-based autotrophic H_2_-dependent growth [21, 79]. In this study, network analysis and prevalence values suggested roles of putative importance for the classes *Betaproteobacteria *, *Gammaproteobacteria* and *Clostridia* in the deep terrestrial subsurface (Fig. 2, Table S3). Their maintained presence in this biome across strikingly dissimilar host-rocks and depth, among others, could be indicative of higher metabolic plasticity, providing physiological advantages over other members of microbial communities.

Lithotrophic microbial metabolisms and mineralogy-driven microbial colonization of relatively inert lithologies have previously been demonstrated with low abundance but more reactive minerals within rock matrices often cited as key controls on community structure [80–83]. Limiting factors for life in the terrestrial deep subsurface such as pressure and temperature are more closely correlated with depth. Growth of bacterial isolates from the deep subsurface has been documented at up to 48 MPa and 50 °C and has been associated with production of extracellular polymeric substances (EPS) [84, 85]. However, robust conclusions on the effects of lithology or depth on the structure and composition of microbial communities across Earth’s crust have presented a widespread challenge for science, as in this study due to the small and varied sample sizes resulting from the contamination-aware filtering process and the limited number of comparable lithology types. Large-scale evidence for the roles of eukaryotes, bacteria, archaea and viruses in the deep terrestrial subsurface and the environmental controls over their occurrence in this biome is still lacking. We recommend a field-level research strategy to gain insights into these aspects of life within Earth’s crust. Larger scale collation of data from samples collected and processed using unified, reproducible workflows will be cognizant of significant potential for contamination and ultimately allow robust insights on wide-ranging microbial metabolic processes in the terrestrial subsurface.

Collecting contamination-free samples from the deep subsurface is difficult but important for cataloguing the authentic microbial diversity of the terrestrial subsurface. This study follows recent recommendations for downstream processing of contaminant-prone samples originated in the deep subsurface (Census of Deep Life project – https://deepcarbon.net/tag/census-deep-life), where physical, chemical and biological, but also *in silico* bioinformatics strategies to prevent erroneous conclusions have been highlighted [38, 86, 87]. This study also follows frequency-based OTU filtration techniques similar to those recommended previously [38] and designed to remove possible contaminants introduced during sampling or during the various steps related to sample processing [38]. The pre-emptive quality control steps hereby undertaken support a non-contaminant origin for the taxa analysed in this dataset. As such, the predominance of typically contaminant taxa affiliated, for example, to the genus *Pseudomonas* is accepted as a representative trend in reflecting the microbial ecology of the terrestrial deep subsurface.

Standardizing sampling, DNA extraction, sequencing and bioinformatics methods and strategies across the subsurface research community would help further reduce methodology-based variations. This would more efficiently permit re-analyses after collection, where methodological variations would be controlled, and robust wide-ranging overarching conclusions would more easily be achieved. Despite this, host-rock matrices and local geochemical conditions often pose unique challenges that require particular protocol adjustments [88]. In the near future, the advent of recently developed techniques for primer bias-free long read 16S rRNA and 16S rRNA-ITS gene amplicon long-read-based sequencing may initiate a convergence of molecular methods from which the deep subsurface microbiology community would benefit greatly [89, 90]. The future of large-scale, collaborative deep subsurface microbial diversity studies should encompass not only an effort towards standardization of several molecular biology techniques but also the long-term archival of samples [91]. Finally, the ecology of domains Eukarya and Archaea across the terrestrial deep subsurface remains generally under-characterized and requires future attention. This study presents an important first step towards characterizing bacterial community structure and composition in the terrestrial deep biosphere.

A global-scale meta-analysis addressing the available 16S rRNA gene-based studies of the deep terrestrial subsurface revealed a dominance of *Betaproteobacteria, Gammaproteobacteria* and *Firmicutes* across this biome. Evidence for a core terrestrial deep subsurface microbiome population was recognized through the prevalence and centrality of the genus *Pseudomonas* (* Gammaproteobacteria *) and several other genera affiliated with the class *Betaproteobacteria *. The adaptable metabolic capabilities associated with the above-mentioned taxa may be critical for colonizing the deep subsurface and sustaining communities therein. The terrestrial deep subsurface is a hard-to-reach, complex ecosystem crucial to global biogeochemical cycles. Efforts by multiple teams of investigators to sequence subsurface ecosystems over the last decade were hereby consolidated to characterize the 12–20 % of global biomass this biome represents [5]. The strict contamination-aware filtering process applied whittled down the publicly available datasets representing terrestrial subsurface bacterial diversity to just 70 samples from two continents, indicating the need for systematic exploration of biodiversity within this major component of the biosphere. As a first step, this study consolidates a global-scale understanding of taxonomic trends underpinning a major component of terrestrial deep subsurface microbial ecology and biogeochemistry.

## Supplementary Data

Supplementary material 1
