# Supplementary material for: A global perspective on bacterial diversity in the terrestrial deep subsurface
Source: Microbiology (Reading). 2023 Jan 17;169(1):001172. doi: 10.1099/mic.0.001172 (PMC9993121; doi:10.1099/mic.0.001172)
Supplement: Supplementary material 1 [file mic-169-1172-s001.pdf]

Supplementary Information – "A global perspective on microbial diversity in the terrestrial deep subsurface"

Authors:

Soares, A.[1,2,3a], Edwards, A.[2,3*], An, D.[4], Bagnoud, A.[5], Bradley, J.[6] Barnhart, E[7,8], Bomberg, M.[9], Budwill, K.[10], Caffrey, S. M.[11], Fields, M.[8,12], Gralnick., J.[13], Kadnikov, V.[14,15], Momper, L.[16], Osburn, M.[17], Mu, A.[18,19,20], Moreau, J.W.[21], Moser, D.[22], Purkamo, L.[9,23,24], Rassner, S. M.[1,3], Sheik, C. S.[25], Sherwood Lollar, B. [26], Toner, B. M.[27], Voordouw, G.[4], Wouters, K.[28], Mitchell, A. C.[1,3*]

Supplementary Text

99% closed-reference OTU analysis

A 99% similarity closed-reference OTU-picking strategy to further minimize potential contamination showed a ~10-fold decrease in the number of OTUs and read numbers (1,065 OTUs and 70,527 reads were left). Further, this reduced the number of retrieved samples to 93.

Following the previously described filtering steps (hold only OTUs represented by more than 500 sequences and present in at least 10 samples), 335 OTUs (67,151 reads) associated to 14 samples were left. For this reduced dataset, 2 OTUs associated to Chloroplast were found, represented by 115 sequences in total. The very reduced number of samples, OTUs an total reads left in the datasset caused the 99% OTU approach to be discontinued.

Supplementary Methodology

Phylogeny of **Pseudomonas** *representative sequences*

Representative 16S rRNA gene sequences for *Pseudomonas* OTUs in the dataset were isolated by retrieving OTU IDs affiliated to this genus in the final dataset using the *subset_taxa* function within *phyloseq*[1]. The SILVA 123 database was then queried against the list of OTU IDs and the results deposited in a FASTA file. Outgroup sequences of genus *Sphingomonas* (Proteobacteria, Alphaproteobacteria, Sphingomonadales, Sphingomonadaceae) were obtained directly from the SILVA database (https://www.arb-silva.de/) and added along with the retrieved *Pseudomonas* 16S rRNA gene sequences to a final FASTA file.

Using MEGA7[2], an alignment was performed using the MUSCLE[3] algorithm and 8 iterations of the UPGMB (combines Neighbour-Joining[4] and UPGMA - Unweighted Pair Group Method with Arithmetic mean) clustering method. An optimal Neighbour-Joining[4] tree (*cf.* **Supplementary Figure 4**) was then created using 500 bootstrapped replicates and the Maximum Composite Likelihood (MCL)[5] method to calculate evolutionary distances.

Supplementary Figures

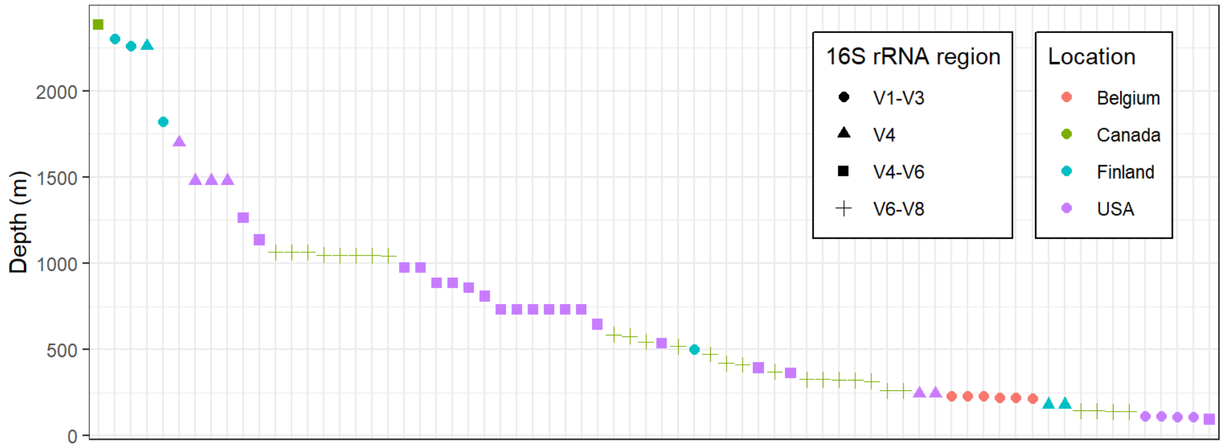

Supplementary Figure 1 – Distribution of samples in the final dataset across depth, colourised by general location. Shapes indicate 16S rRNA gene region utilised for that study.

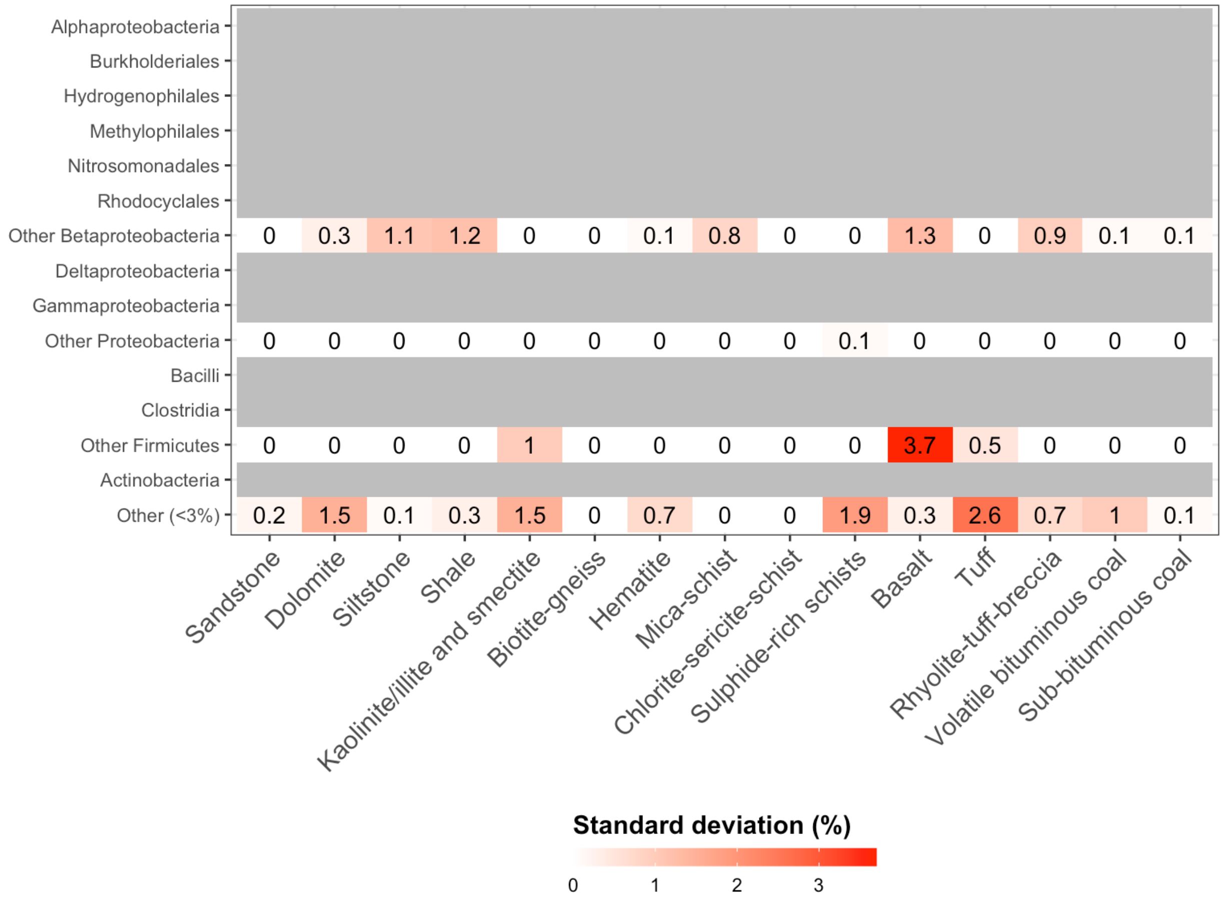

Supplementary Figure 2 – Standard deviations in percentages for **Figure 1**. X-axis shows host-rocks, y-axis depicts the main phyla and classes in the dataset. Grey cells depict non-applicable (NA) standard deviations derived from small sample sizes.

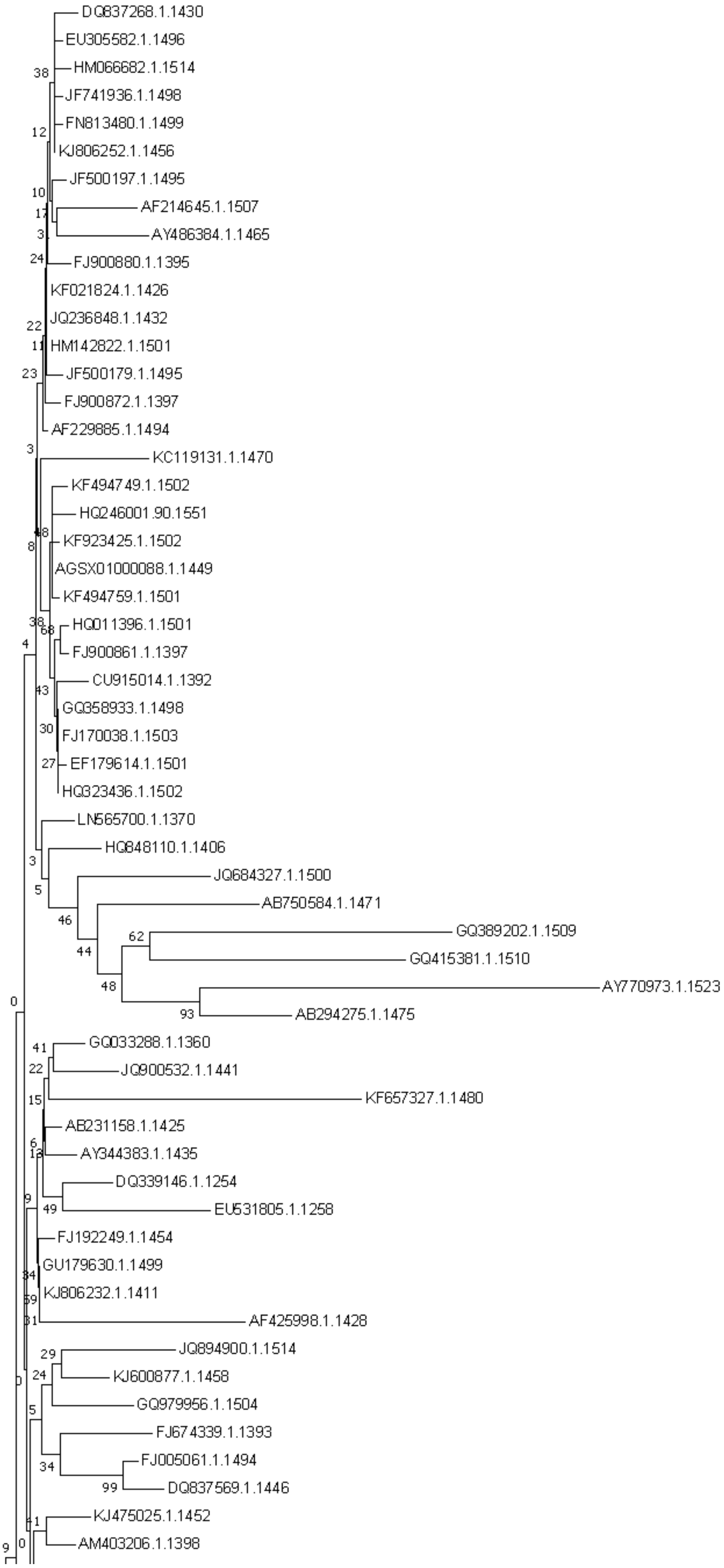
DQ837268.1.1430
EU305582.1.1496
HM066682.1.1514
JF741936.1.1498
FN813480.1.1499
KJ806252.1.1456
JF500197.1.1495
AF214645.1.1507
AY486384.1.1465
FJ900880.1.1395
KF021824.1.1426
JQ236848.1.1432
HM142822.1.1501
JF500179.1.1495
FJ900872.1.1397
AF229885.1.1494
KC119131.1.1470
KF494749.1.1502
HQ246001.90.1551
KF923425.1.1502
AGSX01000088.1.1449
KF494759.1.1501
HQ011396.1.1501
FJ900861.1.1397
CU915014.1.1392
GQ358933.1.1498
FJ170038.1.1503
EF179614.1.1501
HQ323436.1.1502
LN565700.1.1370
HQ848110.1.1406
JQ684327.1.1500
AB750584.1.1471
GQ389202.1.1509
GQ415381.1.1510
AY770973.1.1523
AB294275.1.1475
GQ033288.1.1360
JQ900532.1.1441
KF657327.1.1480
AB231158.1.1425
AY344383.1.1435
DQ339146.1.1254
EU531805.1.1258
FJ192249.1.1454
GU179630.1.1499
KJ806232.1.1411
AF425998.1.1428
JQ894900.1.1514
KJ600877.1.1458
GQ979956.1.1504
FJ674339.1.1393
FJ005061.1.1494
DQ837569.1.1446
KJ475025.1.1452
AM403206.1.1398

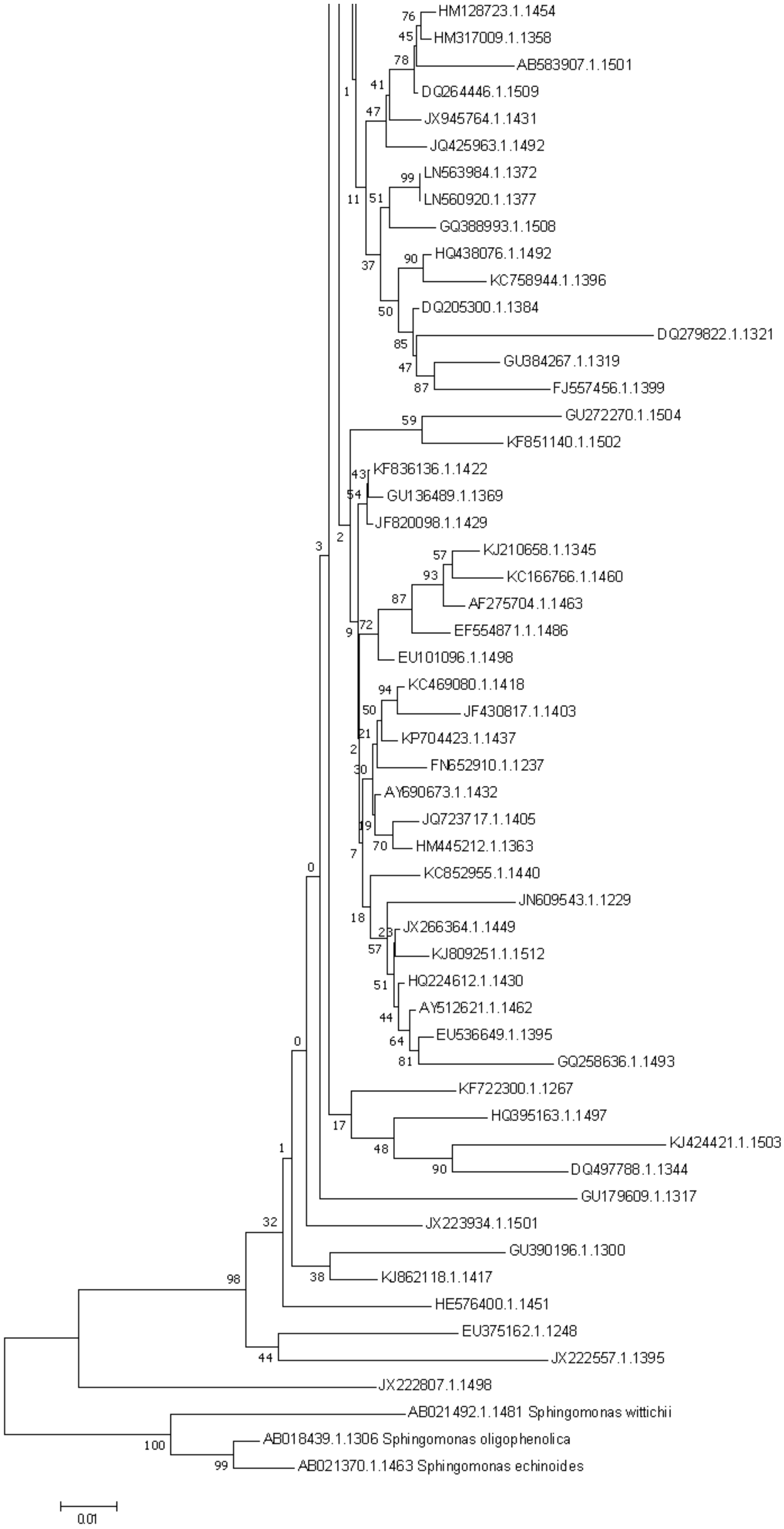
HM128723.1.1454
HM317009.1.1358
AB583907.1.1501
DQ264446.1.1509
JX945764.1.1431
JQ425963.1.1492
LN563984.1.1372
LN560920.1.1377
GQ388993.1.1508
HQ438076.1.1492
KC758944.1.1396
DQ205300.1.1384
DQ279822.1.1321
GU384267.1.1319
FJ557456.1.1399
GU272270.1.1504
KF851140.1.1502
KF836136.1.1422
GU136489.1.1369
JF820098.1.1429
KJ210658.1.1345
KC166766.1.1460
AF275704.1.1463
EF554871.1.1486
EU101096.1.1498
KC469080.1.1418
JF430817.1.1403
KP704423.1.1437
FN652910.1.1237
AY690673.1.1432
JQ723717.1.1405
HM445212.1.1363
KC852955.1.1440
JN609543.1.1229
JX266364.1.1449
KJ809251.1.1512
HQ224612.1.1430
AY512621.1.1462
EU536649.1.1395
GQ258636.1.1493
KF722300.1.1267
HQ395163.1.1497
KJ424421.1.1503
DQ497788.1.1344
GU179609.1.1317
JX223934.1.1501
GU390196.1.1300
KJ862118.1.1417
HE576400.1.1451
EU375162.1.1248
JX222557.1.1395
JX222807.1.1498
AB021492.1.1481 Sphingomonas wittichii
AB018439.1.1306 Sphingomonas oligophenolica
AB021370.1.1463 Sphingomonas echinoides
0.01

Supplementary Figure 3 – Optimal Neighbor-Joining tree of representative sequences for *Pseudomonas* OTUs in the dataset following MUSCLE aligment. OTU IDs are shown in the end of each branch and outgroup sequences of genus *Sphingomonas* identified by their taxonomic affiliation. The percentage of bootstrap test replicates are shown next to tree branches and scale for MCL evolutionary distances is shown in the bottom.

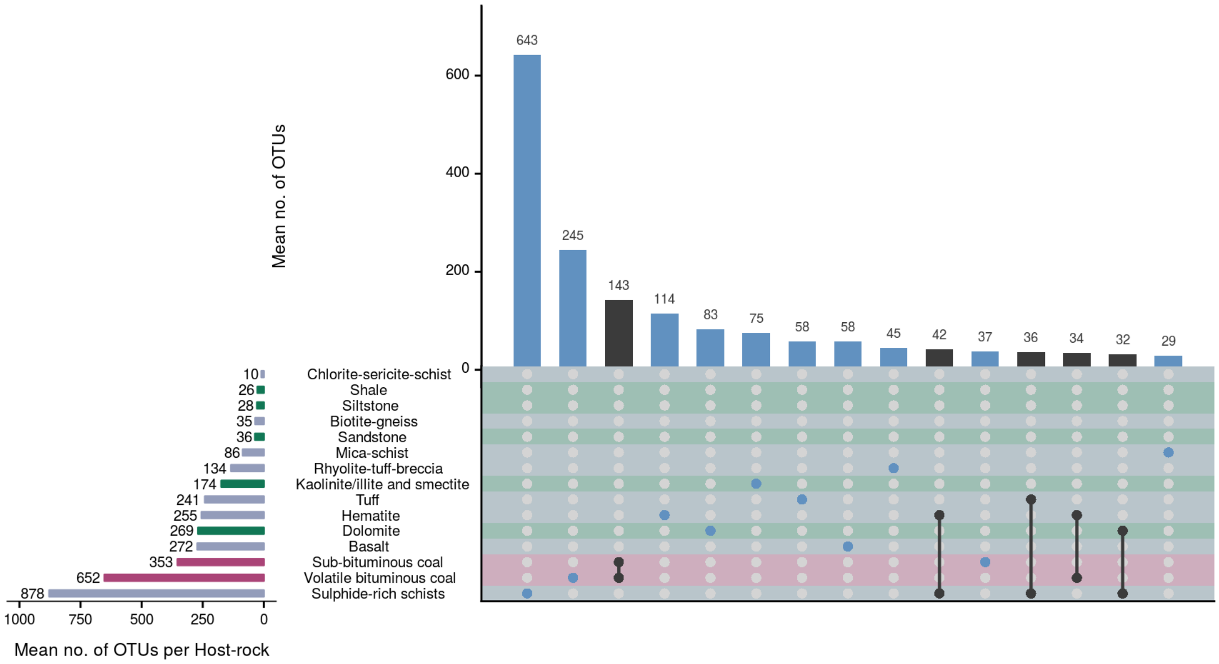

Supplementary Figure 4 - *UpsetR* plot of mean numbers of OTU interactions among rock types. Only interactions involving 25 or more OTUs on average are shown. Coloured matrix rows correspond to host-rocks, and are coloured according to rock type: blue for crystalline, green for sedimentary rocks and pink for coals, which were highlighted due to their higher sample contribution to the dataset. Columns depict OTU interactions: blue dots mark independent (mean number of non-shared OTUs) interactions and black dots connected by black lines mark shared OTUs between two or more host-rocks. Shared interactions are composed of only the host-rocks marked by dots. Vertical bars on top of the coloured matrix correspond to mean OTU numbers present in the described interactions and are coloured black or blue if depicting shared or non-shared interactions, respectively. Horizontal bars by the left of the coloured matrix depict mean total numbers of OTUs per host-rock.

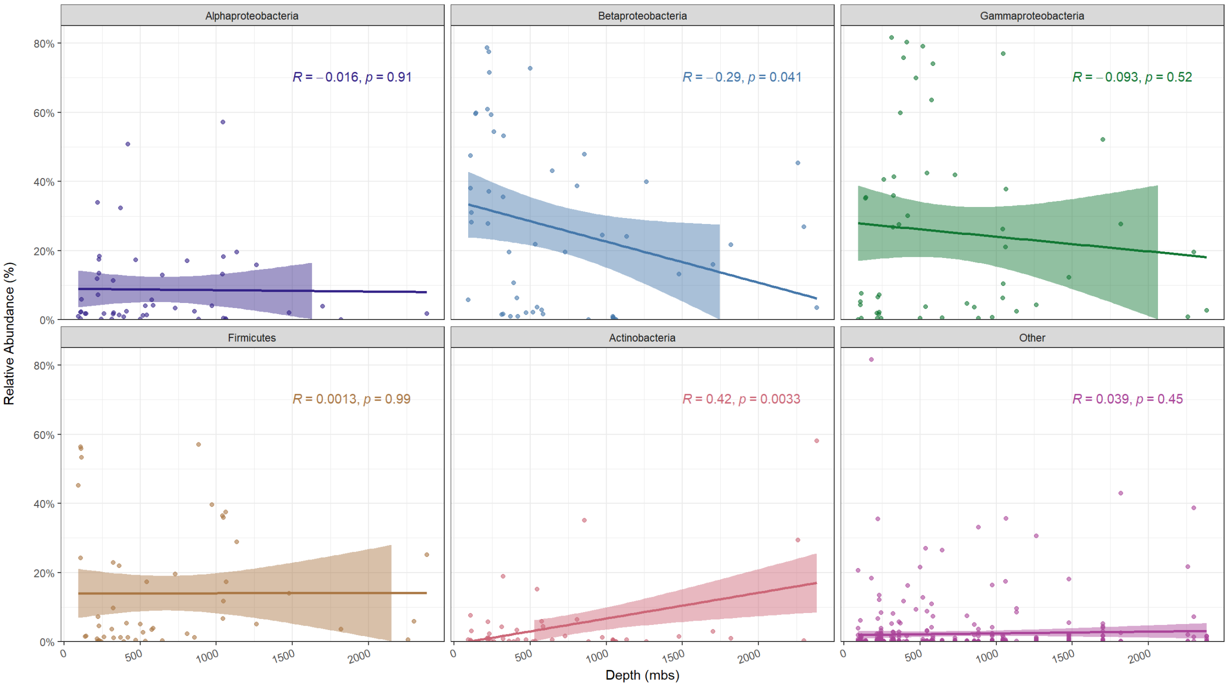

Supplementary Figure 5 - Correlations between relative abundance of OTUs (%, y-axis) associated to the most abundant taxonomic groups across the dataset and depth (meters below surface, x-axis). Regression lines follow the linear model and shading around lines corresponds to the 95% confidence interval. Annotations in plot facets indicate the associated Pearson correlation coefficient, its corresponding p-value and the fitted linear model equation. Each point represents an OTU associated to the taxonomic group in each facet at a certain depth - a same OTU may be depicted more than one time.

Supplementary Tables

Supplementary Table 1 – Numbers of reads following default and the stricter filtering (see **Methodology**) employed in this study across class-level taxonomy.

Class	Default	Stricter
070125-BRIC7-5	99	98
Acetothermia	233	233
Acidobacteria	2222	1942
Actinobacteria	16836	14734
Aerophobetes	3	0
Alphaproteobacteria	35800	26579
Aminicenantes	464	441
Aquificae	3	0
ARKDMS-49	2	0
ARKICE-90	21	11
Armatimonadetes	323	0
Atribacteria	228	143
B1-7BS	10	0
Bacilli	19147	15951
Bacteroidetes	11940	8833
Burkholderiales	70292	61648
Caldiserica	166	161
Candidate division OP3	2059	1998
Candidate division SR1	228	225
Candidate division WS6	58	57
Chlamydiae	20	0
Chlorobi	7667	7557
Chloroflexi	21365	20938
Cloacimonetes	132	99
Clostridia	76415	68695
Cyanobacteria	3394	2546
Deferribacteres	33	20
Deinococcus-Thermus	3679	3644
Deltaproteobacteria	55381	49217
DR-16	3	0
Elusimicrobia	418	384
Epsilonproteobacteria	200	135
Erysipelotrichia	1453	1079
Fusobacteria	9584	9483
Gammaproteobacteria	100724	84278
Gemmatimonadetes	706	630
GOUTA4	3	0
Gracilibacteria	46	30
Hot Creek 32	1375	1232
Hyd24-12	13	13

**Hydrogenedentes**	2276	2260
**Hydrogenophilales**	6583	4972
**JL-ETNP-Z39**	87	84
**JTB23**	10	0
**Kazan-3B-09**	22	12
**Latescibacteria**	1599	1563
**Lentisphaerae**	83	39
**Marinimicrobia (SAR406 clade)**	5	0
**Methylophilales**	4137	3611
**Microgenomates**	164	138
**Negativicutes**	192	102
**Neisseriales**	634	560
**Nitrosomonadales**	52361	51810
**Nitrospirae**	15391	15133
**Omnitrophica**	534	522
**Other (<1%)**	3	0
**Other Betaproteobacteria**	3	0
**Other Proteobacteria**	3	0
**Parcubacteria**	173	150
**PAUC34f**	18	15
**Planctomycetes**	3456	3073
**Rhodocyclales**	11726	9990
**Saccharibacteria**	21	0
**SC-I-84**	16	0
**SC3-20**	2	0
**SHA-109**	1492	1479
**SM2F11**	3	0
**Spirochaetae**	1128	846
**SPOTSOCT00m83**	21	15
**Synergistetes**	63	14
**TA06**	2471	2435
**TA18**	238	222
**Tenericutes**	650	279
**Thermotogae**	64	44
**TM6**	99	81
**TRA3-20**	166	147
**UCT N117**	73	59
**Verrucomicrobia**	2052	1286
**WCHB1-60**	15	15
**WD272**	114	114
**Zetaproteobacteria**	17	0

Supplementary Table 2 – Metadata table with complete details for all studies utilized (CoDL – Census of Deep Life, HMP – Hydrocarbon Metagenomics Project).

SRA Accession	Depth gradient (m)	DNA Extraction method	Sample Type	16S region	Sequencing method	Project
PRJNA262938	243.84-1478.28	Mod. Phenol chloroform	Groundwater	V4	454	
PRJNA268940	94-2383	MoBio UltraClean Microbial DNA isolation kit	Groundwater	V4-V6	454	CoDL
PRJNA248749	730	MoBio PowerWater	Groundwater	V4-V6	454	CoDL
PRJNA251746	393-1135	MoBio_PowerMax_Soil	Groundwater	V4-V6	454	CoDL
PRJNA375701	109-114.7	Mod. MP-Biomedicals FastDNA Spin_Soil [6]	Rock + porewater	V1-V3	454	
NA*	140-1064.4	[7]	Rock + porewater	V6-V8	454	HMP
PRJEB1468	217.13-231.83	XS buffer [8]	Groundwater	V1-V3	454	
PRJEB10822	180-2300	MoBio PowerSoil	Groundwater	V4**, V1-V3	454	

* http://hmp.ucalgary.ca/HMP/

* This primer pair corresponds to archaeal 16S rRNA gene primers (A344,A744, *cf.* 10.5194/bg-13-3091-2016 for further details).

Supplementary Table 3 - SILVA 123 taxonomic affiliations of OTUs present in 20 or more samples. Prevalence is defined as the number of samples an OTU is present in.

Taxa ID	Taxa Classification	Prevalence
JQ236848.1.1432	Proteobacteria, Gammaproteobacteria, Pseudomonadales, Pseudomonadaceae, Pseudomonas	41
HM142822.1.1501	Proteobacteria, Gammaproteobacteria, Pseudomonadales, Pseudomonadaceae, Pseudomonas	34
JX266364.1.1449	Proteobacteria, Gammaproteobacteria, Pseudomonadales, Pseudomonadaceae, Pseudomonas	31
KJ475025.1.1452	Proteobacteria, Gammaproteobacteria, Pseudomonadales, Pseudomonadaceae, Pseudomonas	27
FJ192249.1.1454	Proteobacteria, Gammaproteobacteria, Pseudomonadales, Pseudomonadaceae, Pseudomonas	26
HQ848110.1.1406	Proteobacteria, Gammaproteobacteria, Pseudomonadales, Pseudomonadaceae, Pseudomonas	25
KC852955.1.1440	Proteobacteria, Gammaproteobacteria, Pseudomonadales, Pseudomonadaceae, Pseudomonas	25
HM773515.1.1498	Proteobacteria, Betaproteobacteria, Burkholderiales, Comamonadaceae, uncultured	24
AB231158.1.1425	Proteobacteria, Gammaproteobacteria, Pseudomonadales, Pseudomonadaceae, Pseudomonas	24
JX222276.1.1475	Proteobacteria, Betaproteobacteria, Burkholderiales, Comamonadaceae, Variovorax	23
EU841498.1.1443	Proteobacteria; Gammaproteobacteria; Alteromonadales, Alteromonadaceae, Alishewanella	22
EU305582.1.1496	Proteobacteria, Gammaproteobacteria, Pseudomonadales, Pseudomonadaceae, Pseudomonas	22
FJ900880.1.1395	Proteobacteria, Gammaproteobacteria, Pseudomonadales, Pseudomonadaceae, Pseudomonas	21

Disclaimer: Any use of trade, firm, or product names is for descriptive purposes only and does not imply endorsement by the U.S. Government.

Supplementary Table 4 – BLAST hits table for representative sequences associated to OTUs affiliated to genus *Pseudomonas* .

OTU ID	Closest BLAST hit	Associated NCBI Title	Associated Environment	Taxonomy ID	NCBI Accession	Percent similarity (%)	E-value	No. Samples Associated	No. Reads Associated
AB231158.1.1425	Pseudomonas pseudoalcaligenes gene for 16S rRNA, partial sequence	High nitrogen removal from wastewater with several new aerobic bacteria isolated from diverse ecosystems	Wastewater	330	gi|85539942|dbj|AB231158.1|	100	0	24	157
AB294275.1.1475	Uncultured bacterium gene for 16S rRNA, partial sequence, clone: YWB06	Molecular characterization of microbial communities in deep coal seam groundwater of northern Japan	Subsurface	77133	gi|126143387|dbj|AB294275.1|	100	0	19	135
AB583907.1.1501	Uncultured bacterium gene for 16S rRNA, clone: RBC-4B	Microbial community in water-rock-microbe interaction systems in subsurface environments	Subsurface	77133	gi|324604950|dbj|AB583907.1|	100	0	3	137
AB750584.1.1471	Uncultured bacterium gene for 16S rRNA, partial sequence, clone: 23 hydrate.Cas.16S	Novel integrons and gene cassettes from a Cascadian submarine gas hydrate-bearing core	Subsurface	77133	gi|407969505|dbj|AB750584.1|	100	0	16	69
AF214645.1.1507	Nitrogen-fixing bacterium MIS 16S ribosomal RNA gene, partial sequence	Molecular characterization of plant associated nitrogen-fixing Bacteria	Subsurface	120486	gi|7542430|gb|AF214645.1|AF214645	100	0	4	20
AF229885.1.1494	Pseudomonas sp. 3CB6 16S ribosomal RNA gene, partial sequence	Isolation and characterization of diverse halobenzoate-degrading denitrifying bacteria from soils and sediments	Subsurface	126132	gi|9965646|gb|AF229885.1|	100	0	9	22
AF275704.1.1463	Unidentified Hailaer soda lake bacterium F5 16S ribosomal RNA gene, partial sequence	China: Inner Mongolia	Lake	148462	gi|12275954|gb|AF275704.1|AF275704	100	0	12	47
AF425998.1.1428	Bacterium UNSW3 16S ribosomal RNA gene, partial sequence	A survey of phylogeny and paralytic shellfish poison production from culturable bacteria associated with a toxic Anabaena circinalis strain	Host-associated	190587	gi|23451685|gb|AF425998.1|	100	0	14	56
AGSX01000088.1.1449	Pseudomonas stutzeri SDM-LAC contig000089, whole genome shotgun sequence	Genome Sequence of Pseudomonas stutzeri SDM-LAC, a Typical Strain for Studying the Molecular Mechanism of Lactate Utilization	Lab strain	271420	gi|1093258265|emb|LT629970.1|	99.83	0	6	28
AM403206.1.1398	Pseudomonadaceae bacterium D7-21 partial 16S rRNA gene, isolate D7-21	Diversity of Nitrate-reducing and Denitrifying Bacteria in a Marine Aquaculture Biofilter	Seawater	404904	gi|115334066|emb|AM403206.1|	100	0	8	16
AY344383.1.1435	Unidentified bacterium clone K2-4-3 16S ribosomal RNA gene, partial sequence	Microbial Communities in the Hawaiian Archipelago: A Microbial Diversity Hotspot	Subsurface	1869227	gi|33391921|gb|AY344383.1|	100	0	6	10
AY486384.1.1465	Pseudomonas stutzeri strain AU4823 16S ribosomal RNA gene, partial sequence	PCR-based assay for differentiation of Pseudomonas aeruginosa from other Pseudomonas species recovered from cystic fibrosis patients	Host-associated	316	gi|40019083|gb|AY486384.1|	100	0	7	17
AY512621.1.1462	Pseudomonas veronii strain A1YdBTEX2-5 16S ribosomal RNA gene, partial sequence	Alternative primer sets for PCR detection of genotypes involved in bacterial aerobic BTEX degradation: distribution of the genes in BTEX degrading isolates and in subsurface soils of a BTEX contaminated industrial site	Subsurface	76761	gi|41056888|gb|AY512621.1|	100	0	15	91
AY690673.1.1432	Sulfitobacter sp. GC07 16S ribosomal RNA gene, partial sequence	The diversity of halotolerant heterotrophic bacteria isolated from rhizosphere soil of salt marshes from southwestern coasts in Korea	Subsurface	290364	gi|51243779|gb|AY690673.1|	100	0	1	33
AY770973.1.1523	Uncultured bacterium clone W33 16S ribosomal RNA gene, partial sequence	Molecular analysis of the microbial communities of the oilfield	Subsurface	77133	gi|54695050|gb|AY770973.1|	100	0	10	14
CU915014.1.1392	Pseudomonas stutzeri partial 16S rRNA gene, strain 24a97	marine sediment and soils	Subsurface	77133	gi|239913533|emb|CU915014.1|	100	0	2	13
DQ205300.1.1384	Pseudomonas sp. HI-G1 16S ribosomal RNA gene, partial sequence	Culturable microbial diversity and the impact of tourism in Kartchner Caverns, Arizona	Subsurface	347772	gi|76365589|gb|DQ205300.1|	100	0	2	15
DQ264446.1.1509	Uncultured bacterium clone BANW452 16S ribosomal RNA gene, partial sequence	High-density universal 16S rRNA microarray analysis reveals broader diversity than typical clone library when sampling the environment	Subsurface	77133	gi|82393910|gb|DQ264446.1|	100	0	5	13
DQ279822.1.1321	Pseudomonas sp. D14 16S ribosomal RNA gene, partial sequence	Direct Submission, strain D14	Lab strain	358759	gi|82697883|gb|DQ279822.1|	100	0	6	10
DQ339146.1.1254	Pseudomonas sp. M9J918 16S ribosomal RNA gene, partial sequence	Isolation of glyphosate-resistant bacterial species M9J918 from glyphosate-polluted wheat soil in China	Subsurface	366287	gi|85001608|gb|DQ339146.1|	100	0	9	13
DQ497788.1.1344	Pseudomonas sp. SGb396 16S ribosomal RNA gene, partial sequence	Soil, subusrface, bacterial endophytes from Taxus globosa	Subsurface	77133	gi|98975506|gb|DQ497788.1|	100	0	10	44
DQ837268.1.1430	Pseudomonas stutzeri strain WWvii23 16S ribosomal RNA gene, partial sequence	wastewater in lagos and ogun states Nigeria	Wastewater	86473	gi|112434217|gb|DQ837268.1|	100	0	7	16
DQ837569.1.1446	Pseudomonas sp. K4 16S ribosomal RNA gene, partial sequence	Direct Submission, isolate K4	Lab strain	394945	gi|110704228|gb|DQ837569.1|	100	0	9	18
EF179614.1.1501	Pseudomonas sp. WP02-4-9 16S ribosomal RNA gene, partial sequence	Isolation from deep-sea sediments	Subsurface	444156	gi|148251203|gb|EF179614.1|	100	0	2	4
EF554871.1.1486	Pseudomonas sp. AB42 16S ribosomal RNA gene, partial sequence	Adaptative Potential of Alkaliphilic Bacteria towards Chloroaromatic Substrates Assessed by a gfp-Tagged 2,4-D Degradation Plasmid	Lake	443000;443001;443002;1740285	gi|146747229|gb|EF554868.1|	100	0	9	46
EU101096.1.1498	Uncultured bacterium clone FS0612_B12 16S ribosomal RNA gene, partial sequence	Niche differentiation among sulfur-oxidizing bacterial populations in cave waters	Subsurface	77133	gi|156573149|gb|EU101096.1|	100	0	5	12

Supplementary Table 4 – BLAST hits table for representative sequences associated to OTUs affiliated to genus *Pseudomonas* .

EU305582.1.1496	Uncultured Pseudomonas sp. clone 3-A 16S ribosomal RNA gene, partial sequence	Nitrite removal performance and community structure of nitrite-oxidizing and heterotrophic bacteria suffered with organic Matter	Microcosm	114707	gi|163676410|gb|EU305582.1|	100	0	22	453
EU375162.1.1248	Uncultured Pseudomonas sp. clone Sc13 16S ribosomal RNA gene, partial sequence	Bacterial communities from shoreline environments (costa da morte, northwestern Spain) affected by the prestige oil spill	Coastal water	114707	gi|166407785|gb|EU375162.1|	100	0	2	17
EU531805.1.1258	Pseudomonas pseudoalcaligenes 16S ribosomal RNA gene, partial sequence	Bacterial strains isolated from harvested tiger shrimp (Peneaus Monodon)	Host-associated	330	gi|170962995|gb|EU531805.1|	100	0	7	12
EU536649.1.1395	Uncultured bacterium clone nbt64e03 16S ribosomal RNA gene, partial sequence	A diversity profile of the human skin microbiota	Host-associated	77133	gi|187964754|gb|EU536649.1|	100	0	5	503
FJ005061.1.1494	Pseudomonas sp. enrichment culture clone Guo7 16S ribosomal RNA gene, partial sequence	The bioleaching feasibility for Pb/Zn smelting slag and community characteristics of indigenous moderate-thermophilic bacteria	Subsurface	557865	gi|204342383|gb|FJ005061.1|	100	0	7	17
FJ170038.1.1503	Pseudomonas sp. CF14-10 16S ribosomal RNA gene, partial sequence	sediments of the South China Sea	Subsurface	562724	gi|206585088|gb|FJ170038.1|	100	0	9	568
FJ192249.1.1454	Uncultured Pseudomonas sp. clone GI3-S-5-G03 16S ribosomal RNA gene, partial sequence	Comprehensive census of bacteria in clean rooms by using DNA microarray and cloning methods	Urban	114707	gi|209421869|gb|FJ192249.1|	100	0	26	712
FJ557456.1.1399	Uncultured bacterium clone ET_H_1d10 16S ribosomal RNA gene, partial sequence	Endotracheal tube biofilm inoculation of oral flora and subsequent colonization of opportunistic pathogens	Host-associated	77133	gi|224569195|gb|FJ557456.1|	100	0	6	11
FJ674339.1.1393	Uncultured bacterium clone LL141-1D15 16S ribosomal RNA gene, partial sequence	Synecology of the primary and secondary feedlot habitats of Escherichia coli O157:H7	Biological sample	77133	gi|223678636|gb|FJ674339.1|	100	0	7	10
FJ900861.1.1397	Uncultured bacterium clone C-44 16S ribosomal RNA gene, partial sequence	Comparison of microbial community compositions of injection and production well samples in a long-term water-flooded petroleum Reservoir	Subsurface	77133	gi|229428754|gb|FJ900861.1|	100	0	7	9
FJ900872.1.1397	Uncultured bacterium clone C-55 16S ribosomal RNA gene, partial sequence	Comparison of microbial community compositions of injection and production well samples in a long-term water-flooded petroleum Reservoir	Subsurface	77133	gi|229428765|gb|FJ900872.1|	100	0	9	14
FJ900880.1.1395	Uncultured bacterium clone C-62 16S ribosomal RNA gene, partial sequence	Comparison of microbial community compositions of injection and production well samples in a long-term water-flooded petroleum Reservoir	Subsurface	77133	gi|229428773|gb|FJ900880.1|	100	0	21	326
FN652910.1.1237	Pseudomonas putida partial 16S rRNA gene, isolate P5	Isolation, characterization and screening for antimicrobial activities of Padina pavonica associated bacteria	Host-associated	303	gi|290490824|emb|FN652910.1|	100	0	3	45
FN813480.1.1499	Pseudomonas stutzeri partial 16S rRNA gene, isolate Gr20	The genetic diversity of culturable nitrogen-fixing bacteria in the rhizosphere of wheat	Subsurface	316	gi|295810396|emb|FN813480.1|	100	0	17	30
GQ033288.1.1360	Uncultured bacterium clone nbw1028h09c1 16S ribosomal RNA gene, partial sequence	Topographical and temporal diversity of the human skin microbiome	Host-associated	77133	gi|238303670|gb|GQ033288.1|	100	0	4	10
GQ258636.1.1493	Pseudomonas sp. SR3(2009) 16S ribosomal RNA gene, partial sequence	Direct Submission, strain SR3	Lab strain	659426	gi|256352433|gb|GQ258636.1|	100	0	5	933
GQ358933.1.1498	Pseudomonas sp. BSw21506 16S ribosomal RNA gene, partial sequence	Direct Submission, strain BSw21506	Lab strain	664427	gi|255689465|gb|GQ358933.1|	100	0	12	19
GQ388993.1.1508	Uncultured bacterium clone J41 16S ribosomal RNA gene, partial sequence	Characterization of Bacterial Community Structure in a Drinking Water Distribution System during an Occurrence of Red Water	Subsurface	77133	gi|256593919|gb|GQ388993.1|	100	0	5	11
GQ389202.1.1509	Uncultured bacterium clone D75 16S ribosomal RNA gene, partial sequence	Characterization of Bacterial Community Structure in a Drinking Water Distribution System during an Occurrence of Red Water	Subsurface	77133	gi|256594128|gb|GQ389202.1|	100	0	8	16
GQ415381.1.1510	uncultured bacterium clone DQB-P183 genomic sequence	Microbial diversity in different production waters of Daqing Oilfield	Subsurface	77133	gi|308097127|gb|GQ415381.1|	100	0	10	21
GQ979956.1.1504	Uncultured bacterium clone SRC_NRB027 16S ribosomal RNA gene, partial sequence	Direct Submission, clone SRC_NRB027	Lab strain	77133	gi|261499583|gb|GQ979956.1|	100	0	15	283
GU136489.1.1369	Pseudomonas sp. DY1 16S ribosomal RNA gene, partial sequence	Direct Submission, strain DY1	Lab strain	690347	gi|265263160|gb|GU136489.1|	100	0	7	13
GU179609.1.1317	Uncultured Pseudomonas sp. clone D006011A15 16S ribosomal RNA gene, partial sequence	Microbial diversity profiles of fluids from low-temperature petroleum reservoirs with and without exogenous water perturbation	Subsurface	114707	gi|306491368|gb|GU179609.1|	100	0	2	159
GU179630.1.1499	Uncultured Pseudomonas sp. clone D118231H09 16S ribosomal RNA gene, partial sequence	Microbial diversity profiles of fluids from low-temperature petroleum reservoirs with and without exogenous water perturbation	Subsurface	114707	gi|306491389|gb|GU179630.1|	100	0	9	17
GU272270.1.1504	Pseudomonas amygdali pv. lachrymans str. M301315 chromosome, complete genome	Dynamic evolution of pathogenicity revealed by sequencing and comparative genomics of 19 Pseudomonas syringae isolates	Subsurface	77133	gi|284025723|gb|GU272270.1|	100	0	3	648
GU384267.1.1319	Pseudomonas aeruginosa strain SZH16 16S ribosomal RNA gene, partial sequence	In situ degradation of phenol and promotion of plant growth in contaminated environments by a single Pseudomonas aeruginosa strain	Subsurface	287	gi|288189621|gb|GU384267.1|	100	0	4	13
GU390196.1.1300	Uncultured bacterium clone SLE33F 16S ribosomal RNA gene, partial sequence	Shifts in microbial community structure of granular and liquid biomass in response to changes to infeed and digester design in anaerobic digesters receiving food-processing wastes	Subsurface	77133	gi|312178659|gb|GU390196.1|	100	0	14	70

Supplementary Table 4 – BLAST hits table for representative sequences associated to OTUs affiliated to genus *Pseudomonas* .

HE576400.1.1451	uncultured bacterium partial 16S rRNA gene, clone K16.133 AW	A combined cultivation and cultivation-independent approach shows high bacterial diversity in water-miscible metalworking fluids	Urban	77133	gi|377549755|emb|HE576400.1|	100	0	4	10
HM066682.1.1514	Uncultured bacterium clone EDW07B006_73 16S ribosomal RNA gene, partial sequence	Microbial diversity and impact on carbonate geochemistry across a changing geochemical gradient in a karst aquifer	Subsurface	77133	gi|313770588|gb|HM066682.1|	100	0	8	16
HM128723.1.1454	Uncultured bacterium clone SINN704 16S ribosomal RNA gene, partial sequence	Diversity of bacterioplankton in contrasting Tibetan lakes revealed by high-density microarray and clone library analysis	Lake	77133	gi|295879789|gb|HM128723.1|	100	0	20	184
HM142822.1.1501	Proteobacterium WJQ No.5 16S ribosomal RNA gene, partial sequence	Direct Submission, strain WJQ No. 5	Lab strain	797062	gi|298256144|gb|HM142822.1|	100	0	34	2472
HM317009.1.1358	Uncultured bacterium clone ncd315a03c1 16S ribosomal RNA gene, partial sequence	Temporal shifts in the skin microbiome associated with disease flares and treatment in children with atopic dermatitis	Host-associated	77133	gi|297010604|gb|HM317009.1|	100	0	4	12
HM445212.1.1363	Uncultured bacterium clone BL1289fO5 16S ribosomal RNA gene, partial sequence	Comparison of Bacterial Diversity in Azorean and Hawai'ian Lava Cave Microbial Mats	Subsurface	77133	gi|302398051|gb|HM445212.1|	100	0	7	32
HQ011396.1.1501	Pseudomonas stutzeri strain NCTC10475 genome assembly, chromosome: 1	NCTC10475	Lab strain	77133	gi|307776705|gb|HQ011396.1|	100	0	8	139
HQ224612.1.1430	Pseudomonas sp. SGb387 16S ribosomal RNA gene, partial sequence	Phylogenetic relationships of bacterial endophytes from Taxus Globosa	Host-associated	1248114	gi|411172546|gb|JX897952.1|	99.15	0	15	73
HQ246001.90.1551	UNVERIFIED: Uncultured Pseudomonas sp. 16S ribosomal RNA-like sequence	Proteobacteria dominance in the estuarine belt of river Narmada, India as depicted by molecular phylogenetic approaches	Freshwater	471	gi|226430883|gb|FJ816052.1|	88.52	0	4	19
HQ323436.1.1502	Pseudomonas stutzeri strain M4 16S ribosomal RNA gene, partial sequence	Diversity of airborne bacteria community in the Mogao Grottoes, Dunhuang, China	Air	316	gi|319658795|gb|HQ323436.1|	100	0	6	10
HQ395163.1.1497	Uncultured bacterium clone OTUb51 16S ribosomal RNA gene, partial sequence	Culture-independent and culture-dependent methods reveal diverse bacterial and archaeal communities in a biodegraded Malaysian oil Reservoir	Subsurface	77133	gi|320172034|gb|HQ395163.1|	100	0	4	18
HQ438076.1.1492	Pseudomonas sp. TeU 16S ribosomal RNA gene, partial sequence	Isolation and characterization of an environmental cadmium- and tellurite-resistant Pseudomonas strain	Subsurface	944290	gi|318063761|gb|HQ438076.1|	100	0	8	11
HQ848110.1.1406	Pseudomonas argentinensis strain PL-40-1 16S ribosomal RNA gene, partial sequence	Phylogenetic diversity of culturable bacteria isolated from the disused ancient Kiyik River	Freshwater	289370	gi|340025389|gb|HQ848110.1|	100	0	25	378
JF430817.1.1403	Pseudomonas sp. P56(2011) 16S ribosomal RNA gene, partial sequence	Genetic and functional diversity of fluorescent Pseudomonas from rhizospheric soils of wheat crop	Subsurface	1079824	gi|345132366|gb|JF430817.1|	100	0	14	76
JF500179.1.1495	Uncultured bacterium clone 1572_50bact 16S ribosomal RNA gene, partial sequence	The deep biosphere in terrestrial sediments in the chesapeake bay area, virginia, USA	Subsurface	77133	gi|343170179|gb|JF500179.1|	100	0	11	230
JF500197.1.1495	Uncultured bacterium clone 1563_48bact 16S ribosomal RNA gene, partial sequence	The deep biosphere in terrestrial sediments in the chesapeake bay area, virginia, USA	Subsurface	77133	gi|343170197|gb|JF500197.1|	100	0	10	18
JF741936.1.1498	Uncultured bacterium clone LHJB-8 16S ribosomal RNA gene, partial sequence	Microbial communities in oil reservoirs with different salinities	Subsurface	77133	gi|332712701|gb|JF741936.1|	100	0	3	10
JF820098.1.1429	Pseudomonas sp. PG-3-1 16S ribosomal RNA gene, partial sequence	Diversity of Culturable Butane-oxidizing Bacteria in Oil and Gas Field Soil	Subsurface	1036156	gi|334690415|gb|JF820098.1|	100	0	8	11
JN609543.1.1229	Pseudomonas fluorescens strain Cantas14 16S ribosomal RNA gene, partial sequence	The culturable intestinal microbiota of triploid and diploid juvenile Atlantic salmon (Salmo salar) - a comparison of composition and drug resistance	Host-associated	294	gi|358440878|gb|JN609543.1|	100	0	5	38
JQ236848.1.1432	Pseudomonas stutzeri strain hswx82 16S ribosomal RNA gene, partial sequence	Direct Submission, strain hswx82	Lab strain	316	gi|377830169|gb|JQ236848.1|	100	0	41	5419
JQ425963.1.1492	Uncultured bacterium clone CT0C1CA03 16S ribosomal RNA gene, partial sequence	Bacterial diversity in an alkaline saline soil spiked with Anthracene	Lake	77133	gi|385760667|gb|JQ425953.1|	100	0	9	22
JQ684327.1.1500	Uncultured bacterium clone HWGB-69 16S ribosomal RNA gene, partial sequence	Bacterial and archaeal diversity in permafrost soil from Kunlun Mountains Pass, Tibet Plateau of China	Subsurface	77133	gi|387568220|gb|JQ684327.1|	100	0	3	12
JQ723717.1.1405	Pseudomonas sp. B-5-1 16S ribosomal RNA gene, partial sequence	beach sand in the west sea, Korea	Coastal water	1172620	gi|384392471|gb|JQ723717.1|	100	0	13	68
JQ894900.1.1514	Uncultured bacterium clone YPMB-G12 16S ribosomal RNA gene, partial sequence	Microbial diversity of endosymbiont bacteria in the spiraling Whitefly	Host-associated	77133	gi|411181008|gb|JQ894900.1|	100	0	9	21
JQ900532.1.1441	Pseudomonas mendocina strain B8 16S ribosomal RNA gene, partial sequence	Crude oil degrading bacterial isolates from ecological region of Assam	Subsurface	300	gi|402244732|gb|JQ900532.1|	100	0	2	126
JX222557.1.1395	Uncultured bacterium clone EMIRGE_OTU_s2b2b_12537 16S ribosomal RNA gene, partial sequence	Subsurface microbial community response to acetate amendment	Subsurface	77133	gi|395559126|gb|JX222557.1|	100	0	1	12
JX222807.1.1498	Uncultured bacterium clone EMIRGE_OTU_s3t2d_3949 16S ribosomal RNA gene, partial sequence	Subsurface microbial community response to acetate amendment	Subsurface	77133	gi|395559376|gb|JX222807.1|	100	0	4	9
JX223934.1.1501	Uncultured bacterium clone EMIRGE_OTU_s6b4a_3133 16S ribosomal RNA gene, partial sequence	Subsurface microbial community response to acetate amendment	Subsurface	77133	gi|395560503|gb|JX223934.1|	100	0	5	10
JX266364.1.1449	Pseudomonas sp. B2085 16S ribosomal RNA gene, partial sequence	Depth-Related Changes in Community Structure of Culturable Mineral Weathering Bacteria and in Weathering Patterns Caused by Them along Two Contrasting Soil Profiles	Subsurface	1225045	gi|402549839|gb|JX266364.1|	100	0	31	558

Supplementary Table 4 – BLAST hits table for representative sequences associated to OTUs affiliated to genus *Pseudomonas* .

JX945764.1.1431	Pseudomonas sp. LARP66 16S ribosomal RNA gene, partial sequence	Microbial diversity of Ethiopian soda lakes assessed by cultivation Methods	Lake	1266816	gi|428274238|gb|JX945764.1|	100	0	8	11
KC119131.1.1470	Pseudomonas sp. RCC12 16S ribosomal RNA gene, partial sequence	Isolation of CO2 fixing bacteria from dehradun caves	Subsurface	1268824	gi|429841843|gb|KC119131.1|	100	0	2	9
KC166766.1.1460	Uncultured Pseudomonas sp. clone BC061 16S ribosomal RNA gene, partial sequence	Bacterial community and groundwater quality changes in an anaerobic aquifer during groundwater recharge with aerobic recycled water	Subsurface	114707	gi|523453881|gb|KC166766.1|	100	0	5	15
KC469080.1.1418	Pseudomonas sp. EM174 16S ribosomal RNA gene, partial sequence	Direct Submission, isolate EM174	Lab strain	1282313	gi|452108584|gb|KC469080.1|	100	0	6	19
KC758944.1.1396	Uncultured bacterium clone 12ALLV2e09 16S ribosomal RNA gene, partial sequence	Using DNA-stable isotope probing to identify microorganisms involved in mtbe and tba biodegradation	Coastal water	77133	gi|478444906|gb|KC758944.1|	100	0	2	22
KC852955.1.1440	Pseudomonas cuatrocienegasensis strain LEH6_4A 16S ribosomal RNA gene, partial sequence	Midgut Microbial Community of Culex quinquefasciatus Mosquito Populations from India	Host-associated	77133	gi|523453428|gb|KC852955.1|	100	0	25	293
KF021824.1.1426	Pseudomonas sp. H-144 16S ribosomal RNA gene, partial sequence	Cultured diversity of marine bacteria	Seawater	1345863	gi|513045820|gb|KF021824.1|	100	0	2	9
KF494749.1.1502	Uncultured bacterium clone B24-205 16S ribosomal RNA gene, partial sequence	Vertical changes of the structure of bacterial communities through a permafrost core profile from Qinghai-Tibet Plateau	Subsurface	77133	gi|532529728|gb|KF494749.1|	100	0	2	8
KF494759.1.1501	Uncultured bacterium clone B9-456 16S ribosomal RNA gene, partial sequence	Vertical changes of the structure of bacterial communities through a permafrost core profile from Qinghai-Tibet Plateau	Subsurface	77133	gi|532529738|gb|KF494759.1|	100	0	5	11
KF657327.1.1480	Pseudomonas mendocina strain 2E 16S ribosomal RNA gene, partial sequence	Alkalo Tolerant Bacteria, water	Lake	300	gi|545599206|gb|KF657327.1|	100	0	6	10
KF722300.1.1267	Uncultured Pseudomonas sp. clone DVBSW_J342 16S ribosomal RNA gene, partial sequence	Response of bacterial community structure to seasonal fluctuation and anthropogenic pollution on coastal water of Alang-Sosiya ship breaking yard, Bhavnagar, India	Coastal water	114707	gi|643039831|gb|KF722300.1|	100	0	5	9
KF836136.1.1422	Pseudomonas plecoglossicida strain SBADK2 16S ribosomal RNA gene, partial sequence	rhizosphere bacteria for agricultural and environmental use	Subsurface	70775	gi|578003379|gb|KF836136.1|	100	0	15	46
KF851140.1.1502	Uncultured Pseudomonas sp. clone BJP8S20-c24 16S ribosomal RNA gene, partial sequence	Lithology-Controlled Bacteria Community in an Ammonium-Rich Aquifer-Aquitard System in the Pearl River Delta, China	Subsurface	114707	gi|582054428|gb|KF851140.1|	100	0	10	30
KF923425.1.1502	Pseudomonas xanthomarina strain 15 16S ribosomal RNA gene, partial sequence	Culturable bacteria from the Qinghai-Tibet Plateau	Soil	271420	gi|594591039|gb|KF923425.1|	100	0	8	10
KJ210658.1.1345	Pseudomonas xinjiangensis strain WL-257 16S ribosomal RNA gene, partial sequence	Phylogenetic diversity of eudophytic bacteria from Populus euphratica in 20-year Pupulus jujube forest ecotone of Chakhikh county of Xinjiang	Subsurface	487184	gi|612340510|gb|KJ210658.1|	100	0	8	25
KJ424421.1.1503	Pseudomonas sp. GW28-5 16S ribosomal RNA gene, partial sequence	cytotoxic bacteria from Antarctica	Host-associated	77133	gi|601035987|gb|KJ424421.1|	100	0	4	121
KJ475025.1.1452	Pseudomonas peli strain IARI-RP26 16S ribosomal RNA gene, partial sequence	Prospecting cold deserts of north western Himalayas for microbial diversity and plant growth promoting attributes	Soil	53406;592361	gi|387285851|gb|JQ795777.1|	100	0	27	962
KJ600877.1.1458	Uncultured bacterium clone 83A 16S ribosomal RNA gene, partial sequence	Manipulating the banana rhizosphere microbiome for biological control of Panama disease	Subsurface	77133	gi|646117505|gb|KJ600877.1|	100	0	9	24
KJ806232.1.1411	Pseudomonas alcaligenes strain PBR-49 16S ribosomal RNA gene, partial sequence	Phylogenetic characterization of heterotrophic bacteria isolated from photobioreactor (PBR) cultures of Synechocystis sp. PCC6803	Host-associated	43263	gi|669340641|gb|KJ806232.1|	100	0	10	26
KJ806252.1.1456	Pseudomonas stutzeri strain PBR-57 16S ribosomal RNA gene, partial sequence	Phylogenetic characterization of heterotrophic bacteria isolated from photobioreactor (PBR) cultures of Synechocystis sp. PCC6803	Host-associated	316	gi|669340661|gb|KJ806252.1|	100	0	7	10
KJ809251.1.1512	Uncultured bacterium clone F33GN 16S ribosomal RNA gene, partial sequence	Bacteria associated with arbuscular mycorrhizal fungi within roots of plants growing in a soil highly contaminated with aliphatic and aromatic petroleum hydrocarbons	Subsurface	77133	gi|671777739|gb|KJ809251.1|	100	0	2	40
KJ862118.1.1417	Uncultured Pseudomonas sp. clone 35 16S ribosomal RNA gene, partial sequence	16S rDNA gene clone libraries which isolated from biodesulfurization bioreactor	Microcosm	114707	gi|672443372|gb|KJ862118.1|	100	0	8	79
KP704423.1.1437	Pseudomonas sp. UYFA113 16S ribosomal RNA gene, partial sequence	Identification and characterization of the part of the bacterial community associated with field-grown tall fescue (Festuca arundinacea) cv. SFRO Don Tom s in Uruguay	Subsurface	1605337	gi|756058359|gb|KP704423.1|	100	0	7	11
LN560920.1.1377	Uncultured bacterium partial 16S rRNA gene, clone SIGC587_N11D1_16S_B	Leaf-cutter ant refuse dumps are nutrient reservoirs harboring diverse microbial assemblages	Microcosm	77133	gi|697256444|emb|LN563985.1|	100	0	15	200
LN563984.1.1372	Uncultured bacterium partial 16S rRNA gene, clone SIBS643_N12D0_16S_B	Leaf-cutter ant refuse dumps are nutrient reservoirs harboring diverse microbial assemblages	Microcosm	77133	gi|697256444|emb|LN563985.1|	100	0	6	16
LN565700.1.1370	Uncultured bacterium partial 16S rRNA gene, clone SIBG551_N12D2_16S_B	Leaf-cutter ant refuse dumps are nutrient reservoirs harboring diverse microbial assemblages	Microcosm	77133	gi|697264995|emb|LN565700.1|	100	0	7	123

References

1. **McMurdie PJ, Holmes S**. Phyloseq: An R Package for Reproducible Interactive Analysis and Graphics of Microbiome Census Data. *PLoS ONE* 2013;8:e61217.

2. **Kumar S, Stecher G, Tamura K**. MEGA7: Molecular Evolutionary Genetics Analysis Version 7.0 for Bigger Datasets. *Molecular Biology and Evolution* 2016;33:1870–1874.

3. **Edgar RC**. MUSCLE: multiple sequence alignment with high accuracy and high throughput. *Nucleic acids research* 2004;32:1792–7.

4. **Saitou N, Nei M**. The neighbor-joining method: a new method for reconstructing phylogenetic trees. *Molecular Biology and Evolution* 1987;4:406–25.

5. **Tamura K, Nei M, Kumar S**. Prospects for inferring very large phylogenies by using the neighbor-joining method. *Proceedings of the National Academy of Sciences* 2004;101:11030–11035.

6. **Barnhart EP, Weeks EP, Jones EJP, Ritter DJ, McIntosh JC, *et al.*** Hydrogeochemistry and coal-associated bacterial populations from a methanogenic coal bed. *International Journal of Coal Geology* 2016;162:14–26.

7. **Foght J, Aislabie J, Turner S, Brown CE, Ryburn J, *et al.*** Culturable Bacteria in Subglacial Sediments and Ice from Two Southern Hemisphere Glaciers. *Microbial Ecology* 2004;47:329–40.

8. **Leuko S, Goh F, Ibáñez-Peral R, Burns BP, Walter MR, *et al.*** Lysis efficiency of standard DNA extraction methods for Halococcus spp. in an organic rich environment. *Extremophiles* 2008;12:301–308.
